# 

**Published:** 1897-10-23

**Authors:** 


					THE HOSPITAL. ABSOLUTELY PURE, therefore BEST. Oct. 23rd, 1897. ,
CADBURY'S rv w- CADBURY'S
COCOA iNi J*8 cocoa
NO ALKALIES USED.
ORiVICH HOSP.i.TAJ.
No. 578. Vol. XXIII. [J3?EJ?] Saturday,
Oct. 23rd, 1897.
[SubscnpUon Tcnns.j pRICE TWOPENCE.

				

